# Supplementary material for: The HMGB1-RAGE Axis Drives the Proneural-to-Mesenchymal Transition and Aggressiveness in Glioblastoma
Source: Int J Mol Sci. 2025 Sep 25;26(19):9352. doi: 10.3390/ijms26199352 (PMC12524595; doi:10.3390/ijms26199352)
Supplement: Supplementary file 1 [file ijms-26-09352-s001.zip › ijms-3799339-supplementary.pdf]

Supplementary Table, Figures and Western blot raw data

The HMGB1-RAGE Axis Drives the Proneural-to-Mesenchymal Transition and Aggressiveness in Glioblastoma

Hao-Chien Yang [1,2,3], Yu-Kai Su [1,2,3,4], Vijesh Kumar Yadav [5], Iat-Hang Fong [1,2,3,4], Heng-Wei Liu [1,2,3,4,*] and Chien-Min Lin [1,2,3,4,*]

[1] Division of Neurosurgery, Department of Surgery, Taipei Medical University–Shuang Ho Hospital, New Taipei City 23561, Taiwan

[2] Department of Neurology, School of Medicine, College of Medicine, Taipei Medical University, Taipei City 11031, Taiwan

[3] Taipei Neuroscience Institute, Taipei Medical University, Taipei 11031, Taiwan

[4] Graduate Institute of Clinical Medicine, College of Medicine, Taipei Medical University, Taipei City 11031, Taiwan

[5] Department of Medical Research & Education, Taipei Medical University–Shuang Ho Hospital, New Taipei City 23561, Taiwan

* Correspondence: henryway0404@tmu.edu.tw (H.-W.L.); m513092004@tmu.edu.tw (C.-M.L.);
Tel.: +886-2-29307930 (ext. 6942) (H.-W.L.); +886-2-2490088 (ext. 8889) (C.-M.L.);
Fax: +886-2-2248-0900 (H.-W.L.); +886-2-2248-0900 (C.-M.L.)

Table S1: CCLE transcriptomic comparison of proneural-leaning U87-MG versus mesenchymal-like GBM8401 glioblastoma cell lines

Gene	*Functional role / subtype marker*	*U87-MG(log₂ TPM, CCLE 24Q1)*	*GBM8401(log₂ TPM, CCLE 24Q1)*
OLIG2	proneural lineage TF	**7.8**	3.2
PDGFRA	PN receptor tyrosine kinase	**6.4**	2.1
SOX2	stemness / PN TF	**8.1**	4.5
CD44	MES surface marker	2.4	**9.3**
CHI3L1 (YKL-40)	MES secreted glycoprotein	1.9	**8.7**
VIM	MES cytoskeletal protein	4.5	**8.2**
REL	NF-κB family, MES-biased	3.7	**6.9**
MGMT	DNA-repair enzyme	**7.2** (part-methylated)	1.8 (unmethylated)

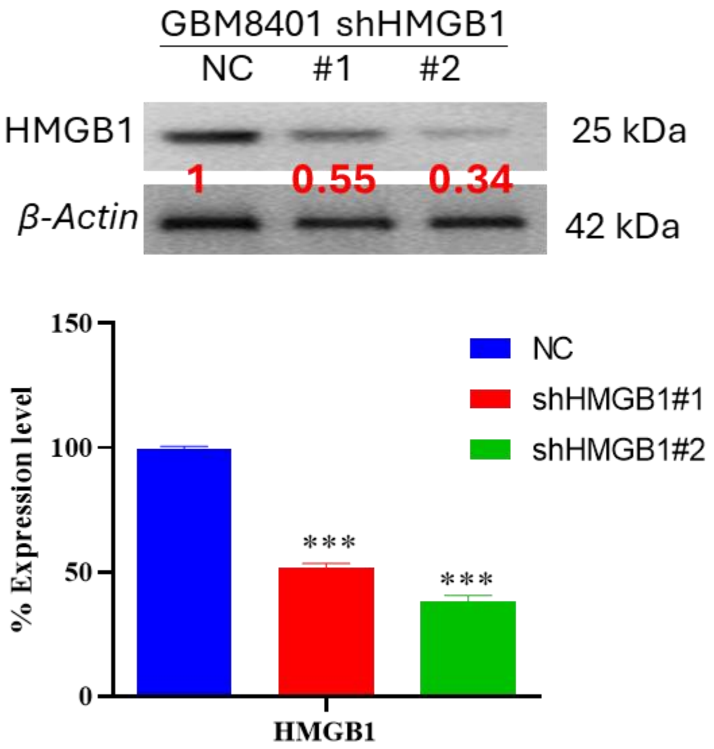

Figure S1: HMGB1 knockdown efficiency and quantification.

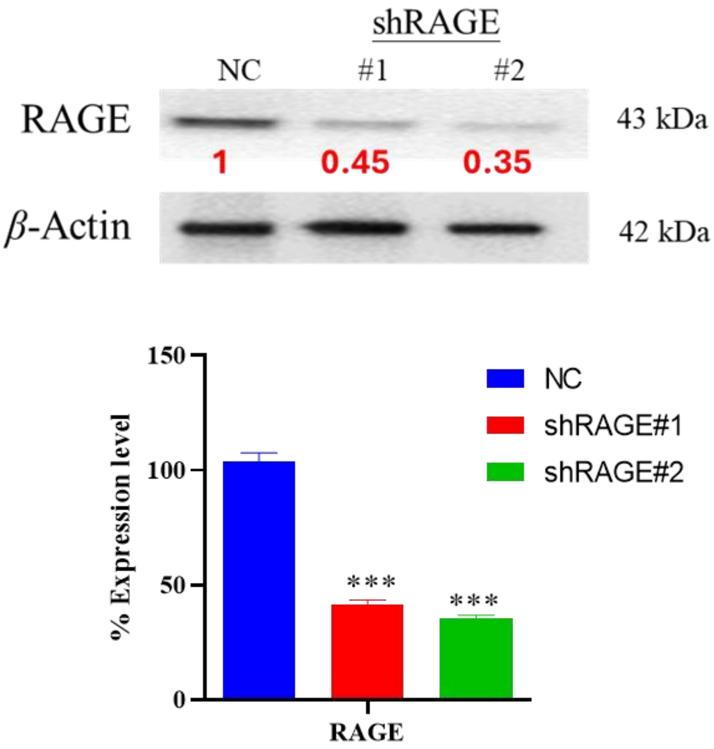

Figure S2: RAGE knockdown efficiency and quantification.

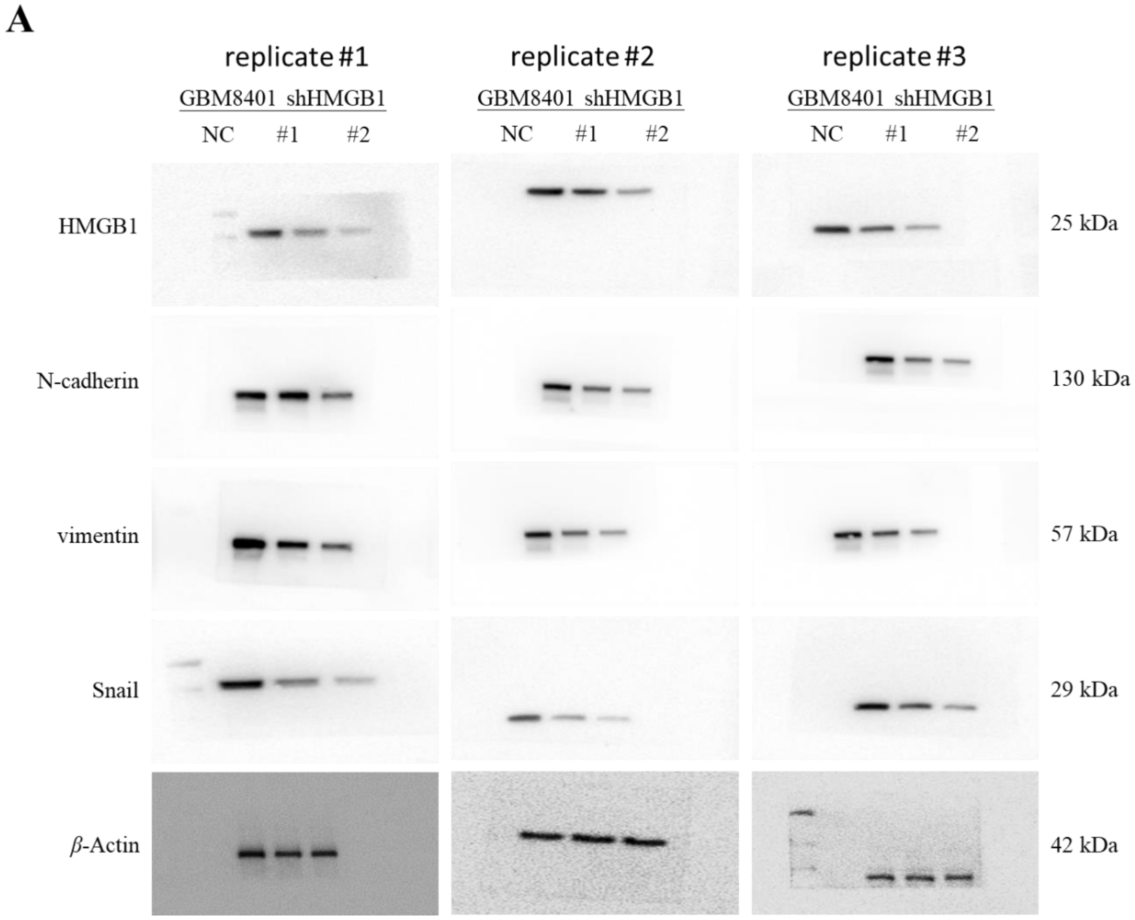

Supplementary Figure S3. Three replicates with Full-size blots of **Figure 3C**

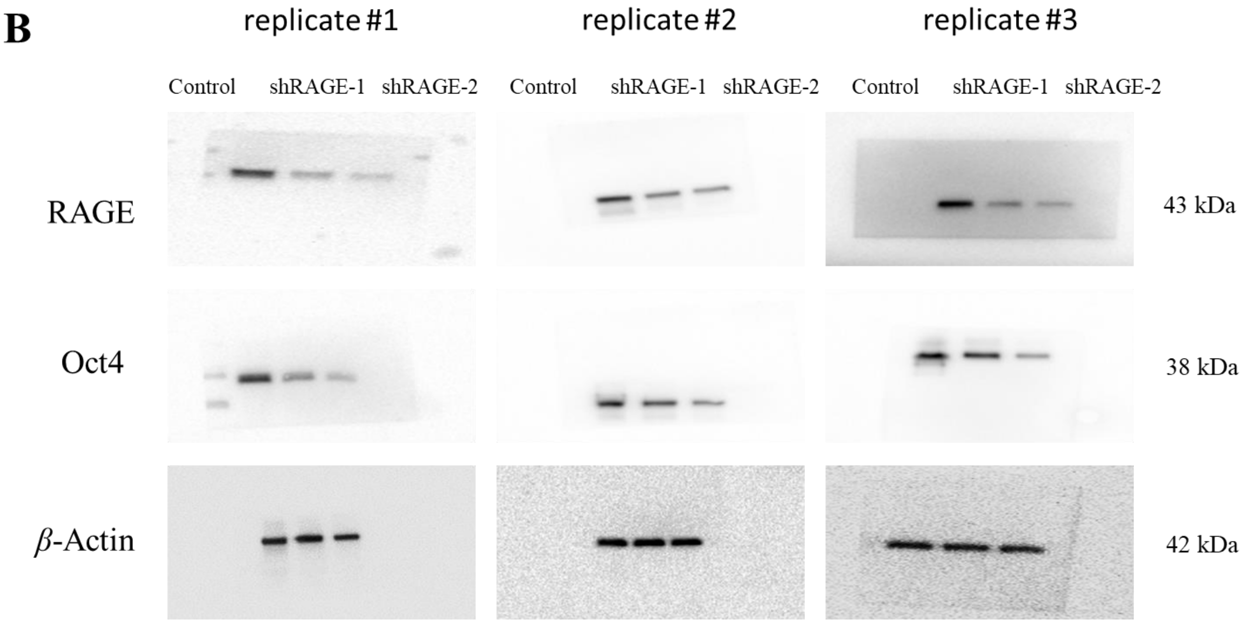

Supplementary Figure S4: Three replicates with Full-size blots of **Figure 6B**.
